# Removal of Nanoparticles by Surface Nanobubbles Generated via Solvent–Water Exchange: A Critical Perspective

**DOI:** 10.1021/acs.langmuir.4c02862

**Published:** 2024-12-16

**Authors:** Pierluigi Bilotto, Daniela Miano, Alper Tunga Celebi, Markus Valtiner

**Affiliations:** † CEST GmbH, Centre for Electrochemical Surface Technology, A-2700, Wiener Neustadt, Austria; ¶ Applied Interface Physics, TU Wien, A-1040, Vienna, Austria

## Abstract

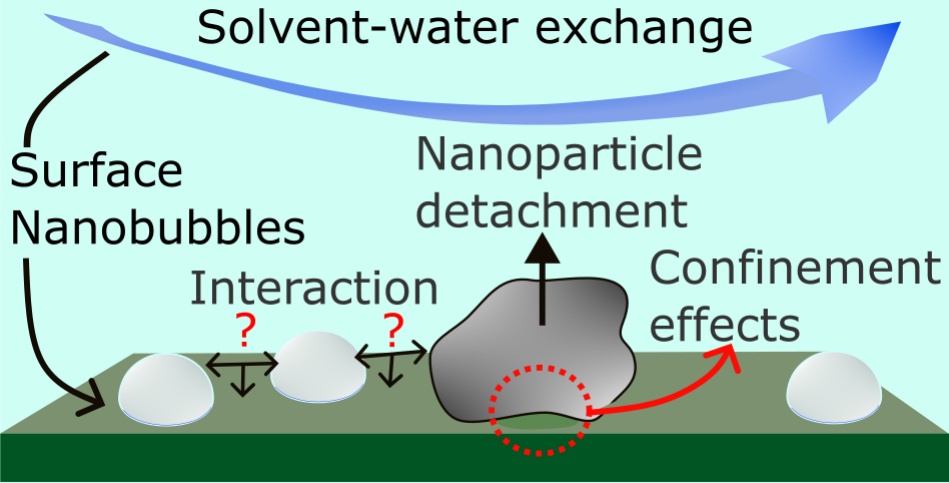

The swift progression of technology in electronic fabrication is adhering to a trend of miniaturization, descending to the nanoscale. Surface contaminants, such as nanoparticles, can influence the performance of silicon wafers, thereby necessitating the evolution of novel cleaning methodologies. Surface nanobubbles (SNs) are phenomena that have attracted considerable attention over the past decade. A salient feature of SNs is their capacity to eliminate nanoparticles from silicon wafers. In this Perspective, our objective is to scrutinize whether this capability can be unequivocally ascribed to SNs. Initially, we offer a succinct elucidation of the nature of SNs; subsequently, we evaluate the claims regarding the cleaning efficacy of SNs; finally, we present our interpretation of the operative forces and propose potential scenarios of the interaction between SNs and nanoparticles. Consequently, the aim of this Perspective is to emphasize the significance of comprehending the interaction between SNs and nanoparticles with the intent to delineate new research trajectories bearing both fundamental and industrial ramifications.

## Introduction

Miniaturization, a prevailing trend in the manufacturing sector, is characterized by the production of increasingly smaller mechanical, optical, and electronic devices. This trend has been propelled by diverse markets, including but not limited to aerospace, media, energy, medical, and electronic industries, where semiconductor materials are heavily used. The global semiconductor market is projected to reach more than 700 billion USD by 2027. Parallel to this trend, cleaning technologies have evolved to secure particle-free, i.e., better performing, wafers.^1^ However, as devices continue to approach the nanoscale following the miniaturization trend, new challenges in cleaning technologies arise, resulting in nanoparticles (NP) having more impact on device efficiency compared to previous technologies (micronscale). Indeed, the presence of NPs (objects with at least one dimension below 100 nm) can deteriorate the electronic performance of miniaturized devices and may even cause short circuits. It is therefore imperative to understand how to remove NPs consistently with high efficiency.

The key to particle removal is to promote detachment by utilizing external stimuli or by moderating adhesive interactions. For instance, Marangoni drying, which consists of promoting mass transfer at interfaces by imposing a gradient in surface tension,^2^ has been extensively used in semiconductor industry, also in combination with solution spinning (Rotagoni method).^1^ Nevertheless, this method is strongly limited by variations of surface properties (e.g., wettability), and it is not efficient in removing particles with nanometer dimensions.^3^ Megasonic cleaning, which exploits acoustic waves to start cavitation of micrometer-scale bubbles, considerably promotes particle removal efficiency (PRE). The method has been employed to remove nanoparticles, albeit it has been shown that it may lead to distributed damages on the wafer.^4^ A further approach is to excite nanoparticles with a laser (or plasma) to exploit the shock-wave propagating underneath the particles acting against the adhesion force.^5^ Similarly to the mega-sonic cleaning, laser-induced cleaning damages the substrate by creating pits on the surface due to the local high pressure at the nanoparticle/wafer interface.^6^ Solvent water exchange (SWE) has demonstrated a significant particle removal efficiency (PRE) in semiconductor wafer cleaning processes.^7^ However, mechanistic details of what promotes particle detachment require nanoscale investigations.

In 2011 the SWE process was tested at the nanoscale on silicon wafer, demonstrating PRE up to 90%.^8^ Nanoparticles were removed without damaging the wafer surface, effectively surpassing the limitation of acoustic or laser cleaning discussed above. The proposed cleaning mechanism consisted of promoting the formation of surface nanobubbles (SNs) by SWE which will then affect the intermolecular forces anchoring NPs to the surface (S). The forces governing bubble-particle interaction at the micronscale have been discussed in in the last 20 years with major breakthroughs in understanding electrostatic, van der Waals (VdW), hydrophobic, and other non-DLVO interactions.^9−12^ SNs generated by SWE interacting with particle have been studied with atomic force microscopy (AFM), with the tip mimicking the particle and revealing nanomechanical properties of SNs such as friction and adhesion.^13^ However, a three-bodies system such as the one formed by SNs-NPs-S presents an additional level of complexity and, to the best of our knowledge, has not been well characterized yet.

In this perspective, we aim to shed light on what has been discussed so far in the literature regarding SNs generated by SWE and their ability to remove NPs attached onto S.

In the first section, we give an overview of what SNs are and how we can assert that they exist. In the second section, we will critically discuss the role of SNs on the wafer cleaning process with respect to the current understanding in the literature. Finally, in the last section, we present our standpoints of surface cleaning mechanism with SNs, and we formulate fundamental open questions and technological challenges.

## What Are Surface Nanobubbles?

**Figure 1:**
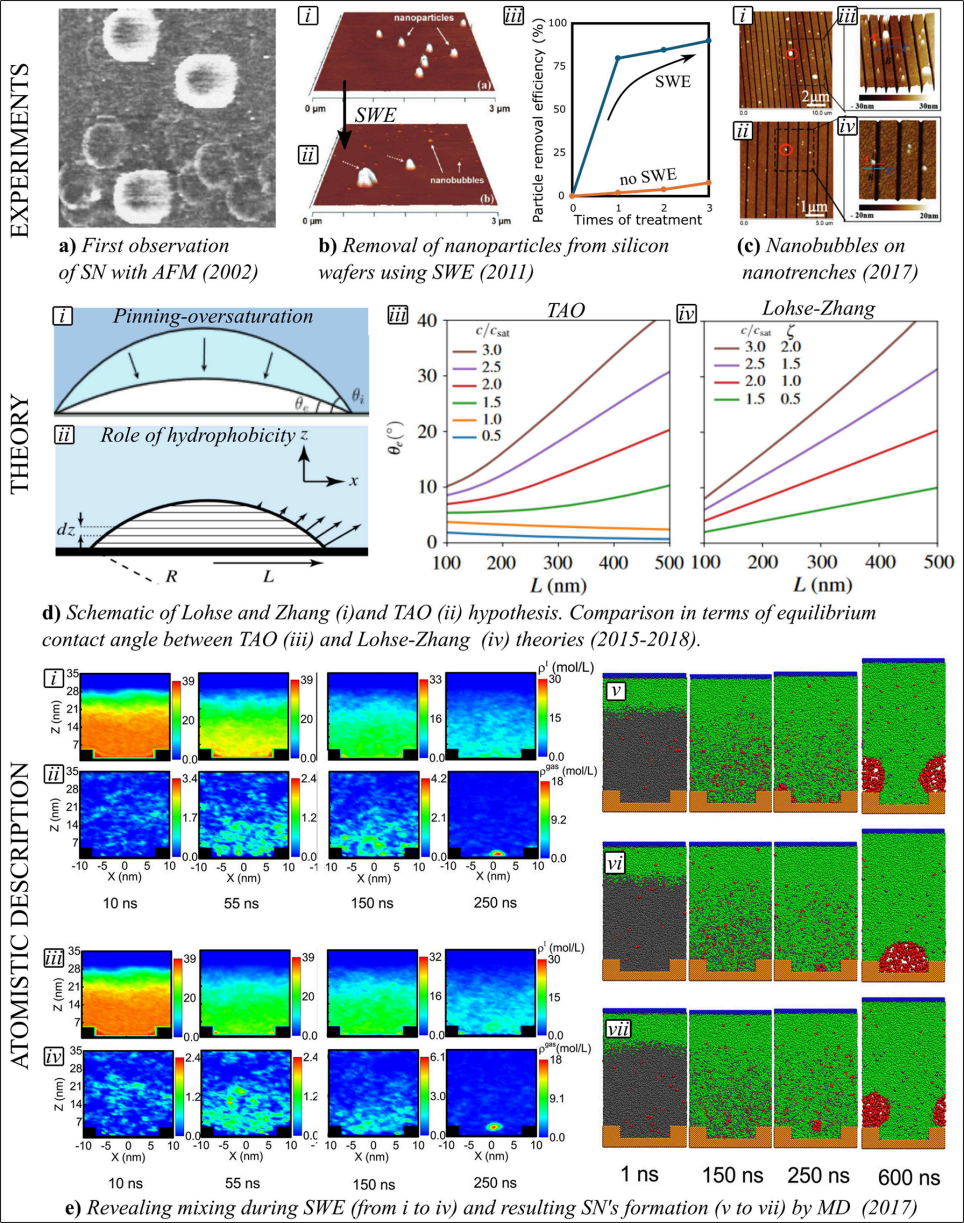
a) Observation of SNs with AFM. Adapted with permission from ref (40). Copyright 2002 Elsevier. b) First demonstration of SNs ability to remove nanoparticles with SWE in AFM (i,ii) and obtained PRE (iii). Adapted with permission from ref (8). Copyright 2011 American Chemical Society. c) Accumulation of SNs on nanotrenches. Adapted with permission from ref (35). Copyright 2017 RSC Pub. d) SN schematic in Lohse-Zhang (i). Adapted with permission from ref (22). Copyright 2015 American Physical Society. SN schematic in the TAO model (ii) and comparison of equilibrium contact angle between TAO (iii) and Lohse-Zhang theory (iv). Adapted with permission from ref (32). Copyright 2018 American Physical Society. e) Density profiles of molecules during SWE on substrates with contact angles 91° (i,ii) and 31° (iii,iv). The density profiles for solvent (i,iii) and gas molecules (ii,iv) are shown at different time during SWE. Nucleation of SNs on substrates of different wettabilities (v-vii): the results is the same although the nucleation pathways differ. Adapted with permission from ref (37). Copyright 2017 American Chemical Society. This publication is licensed under the CC-BY-NC-ND.

Nanobubbles are classified as gas/vapor phase objects in an aqueous solution presenting at least one dimension in the nanoscale (lower than 100 nm). Bulk nanobubbles have been well characterized in the last decades to a point that now they are used in industry.^14,15^ In contrast, SNs are still not industrially exploited due to the complexity of their interaction with surfaces.^16^Figure 1 presents selected works on SNs-S interaction leading to the evidence of SNs growth and stability.

### Experimental Evidence

There are many methods to generate SNs such as microwave irradiation,^17^ water electrolysis,^18^ and SWE. The latter has granted many physical insights on the nature of SNs as it can be easily coupled with AFM. During SWE, a solvent (e.g., alcohol) is substituted by water on a surface. The variations in solubility, surface tension, and the supersaturation of dissolved gases result in the formation of SNs.

One of the first evidence of the formation of SNs by SWE was delivered in the early 2000s by Lou et al.^19^ SNs were produced on a hydrophobic highly oriented pyrolytic graphite using SWE, and monitored using AFM in tapping mode, as shown in Figure 1a. Phase shift analysis during AFM topography (phase shift approximately 50°) and AFM force spectroscopy were employed to distinguish soft (i.e., SNs) from hard (i.e., NPs or contaminants) objects.^13,20^ Furthermore, the presence of SNs was validated over surfaces of different wettability.^20^ Maximum height profiles of SNs were found in a range from 10 to 100 nm, while the width profiles could vary between 100 to 1000 nm depending on experimental parameters (e.g., temperature, pressure).^21^ Such height vs width ratio suggests that the ideal geometrical description of a SN is a spherical cap-shaped surface.^22^ These experimental efforts to view and characterize SNs have simultaneously enabled researchers to develop theoretical models capturing the shape and stability of SNs.

### Theoretical Effort

#### The Surface Nanobubble Paradox

Epstein and Plesset described in 1950 the full diffusive dynamics of a spherical bubble of radius *R*_0_ (any size) by coupling the diffusion equation, the Laplace pressure, and the Henry’s law.^23^ The lifetime of the bubble τ_life_ can be calculated as a function of the gas concentration far away from the bubble *c*_∞_ and the gas solubility *c*_*s*_. τ_life_ is proportional to the square of *R*_0_, which is inversely proportional to Laplace’s pressure. Thus, eq 1 describes that for extremely small *R*_0_, τ_life_ converges to an infinitesimal value due to the extremely high Laplace pressure building up inside the bubble.
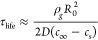
1Later in 1997, Ljunggren and Eriksson calculated the lifetime of a nanobubble as τ_life_ ≃10^–6^ s using eq 1.^24^ The obtained result was strongly in contrast with the experimental value, where SNs were found stable in time (scale of minutes and hours).^16^ This discrepancy started the so-called *surface nanobubble paradox*.^25^

Density functional theory (DFT) and kinetic lattice DFT represented the first attempt to describe the thermodynamic metastable state of SNs, introducing the concept of contact line pinning as key enabler of nanobubble’s stability.^26^ Then, Lohse and Zhang proposed the pinning-oversaturation theory, solving the paradox.^16,22^ They highlighted two crucial points: first, that the Epstein and Plesset formulation is not adequate to describe SNs as it does not involve surfaces; second, that the equilibrium radius of curvature *R*_e_ and the equilibrium contact angle θ_e_ are stabilized by the gas oversaturation ζ:
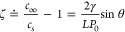
2where *L* is the SN’s footprint radius, θ is the gas-side contact angle, *P*_0_ is the atmospheric pressure, and γ is the surface tension. Due to the pinning process, the initial contact angle θ reduces to an equilibrium contact angle (θ_e_). Figure 1d shows the relation between θ_e_ and *L* according to the Lohse-Zhang model. The role of ζ is to press gas into the bubble from the bulk to balance the Laplace pressure during interface equilibration. Figure 1d)iv shows that for ζ > 0, the higher the ζ, the faster is the gradient of θ_*e*_ for different SN’s footprints, granting the overall stability.

#### Tan-An-Ohl Theory - TAO

An important limitation of the pinning-oversaturation model was its inability to predict the stability of SNs in open systems and undersaturation conditions (), as observed in experiments.^27,28^ The Tan-An-Ohl theory (TAO) expands the pinning-oversaturation model by taking inspiration from the description of intermolecular forces acting at short distances *z*. In the first nanometers away from the substrate, short-range forces such as electric double layer (EDL) repulsion or VdW attraction become predominant. Short range forces have been investigated extensively in the last decades, especially with techniques such as the surface forces apparatus (SFA) and AFM.^29−31^ Specifically, with SFA was demonstrated that substrates possess a short-range hydrophobic potential ϕ (*z*) = ϕ_0_*e*^–*z*/λ^, where λ is the characteristic decay length.^31^ The TAO model employs short-range forces to describe real systems where ζ cannot be defined as homogeneous throughout the liquid. Specifically, a hydrophobic potential must be included to act on the spatial distribution of the gas layer adjacent to the solid substrate.^32^

Consequently, the spherical cap description in Figure 1d)i, is expanded to a cap cut into vertical slices defining ζ(*z*), with each cut having an infinitesimal height *dz* (see Figure 1d)ii). If the substrate potential is ϕ(*z*), the diffusive transport of a liquid layer of thickness *l* needs to include this perturbation as follows:
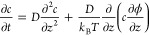
3where *D* is the coefficient of diffusion. Then, eq 3 can be solved for the hydrophobic potential ϕ (*z*):
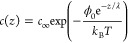
4Therefore, λ can be interpreted as a layer of gas reservoir at the nanobubble-substrate interface that helps the stabilization by expanding θ_*e*_.

For ϕ_0_ = −2*k*_*b*_T, the equilibrium contact angle θ_*e*_ as a function of *L* exists even for undersaturation conditions . (Note that here is utilized *c*(*z*) instead of *c*_∞_, but the former converge to the latter in case of no hydrophobic potential, that is Lohse-Zhang.) Figure 1d)iii illustrates this concept, sided with the Lohse-Zhang output (Figure 1d)iv). Moreover, for small *L*, θ_e_ is smaller in the Lohse-Zhang theory. This is because the TAO model predicts θ_e_ increasing with the localized oversaturation surrounding the nanobubbles and driven by hydrophobic forces that collaborate to the formation of the thin gas reservoir. As soon as *L* increases, the two models converge since λ becomes negligible compared to the bubble height.

To conclude, the most relevant result of the TAO model is the prediction of nanobubbles surviving in degassed liquids because of the stability of the supersaturated film even in bulk-undersaturated conditions.^32,33^

### Atomic Description by Simulations

A common point of confusion lies in distinguishing between the concepts of nanodroplets and nanobubbles. A nanodroplet consists of liquid molecules, while nanobubbles are composed of gas molecules. An excellent discussion on nanobubbles and nanodroplets is presented in an earlier review,^16^ and will not be detailed here. The key point is that the formation of nanobubbles or nanodroplets highly depends on their atomic characteristics. Thus, we hereby discuss the role of gas concentration of the SNs given that the molecular species that compose air are directly related to the growth and stability of SNs generated from SWE. The solubility of air (approximated to nitrogen) in water, at standard temperature and pressure, is approximately 0.02 g/L and governed by Henry’s law. Alcohols such as isopropyl alcohol are highly soluble in water due to −OH termination forming hydrogen bonds. Their exact solubility depends also on the temperature, pressure, and intermixing within the water reservoir. This interplay is mainly controlled by molecular forces between the atomic species. Therefore, an atomistic description obtained by computational approaches, such as molecular dynamics (MD) simulations, can provide further details on the SWE process.

MD simulations accompanied SNs research on growth and stability,^34^ role of wettability,^35^ and the effect of gas species on θ_e_.^36^ The key advantage of MD simulations is that they can simulate the dynamic formation of SNs, which is currently not possible in experiments. Starting from the gas evolution during SWE, it has been shown the actual solvent exchange process in terms of density of dissolved solvent and gas on substrates of different wettability (Figure 1e)i-iv). MD could visualize on one side the nucleation steps that ended in nanobubbles formation on surface of different wettabilities (Figure 1e)v-vii), on the other side the interplay between gas and solvent molecules, which defined a solvent–solvent interface.^37^ Beyond diffusion, MD simulations provided insights into the effective interaction potential between solvated gas molecules and a planar substrate to appreciate the parameters that influence the stability and characteristics of SNs. By expanding the TAO model, substrate wettability, gas affinities in undersaturated conditions, and gas destabilization in organic solvents, could be investigated and successfully compared to experimental findings.^38^

To summarize, the SNs formation and stability can be addressed with experimental, theoretical, and atomistic efforts. If stability has reached a level of maturity, nucleation is still puzzling researchers. For instance, one of the most recent hypotheses (2024), highlights the role of hydrocarbon layers. While being promoted toward adsorption on the interface, they generate a gas enriched layer.^39^ Then, when the water flux flushes along the surface, the layer acts as a platform while the dynamics of gas and water molecules catalyze SNs’ formation. The great challenge here remains the limited experimental accessibility to real-time visualization of the nucleation process.

## Surface Nanobubble-Nanoparticle Interaction as a Cleaning Process

Intermolecular forces drive the interaction between nanobubbles and NPs.^13^ For bulk nanobubbles, the surface charge establishing at their interface promotes aggregation of NPs with positive or negative charge. Consequently, flotation can be initiated and controlled with large scope in cleaning technologies.^41^

However, the current understanding of bulk nanobubble–NPs interaction does not provide sufficient details on the intermolecular forces driving the SNs-NP-S system, calling for further research activities in the field.

### Surface Nanobubbles and Cleaning Efficiency

The nucleation, growth, and dynamics of SNs affect the stability of particles adhered to a surface. Different formation mechanisms underpin distinct processes of particle detachment. For instance, SNs generated by electrolysis emerge from the electrode’s interface (i.e., S), promoting NPs detachment.^42^ This cleaning mechanism relies on the uniform growth and distribution of SNs across the electrode’s interface. In contrast, SWE involves the exchange of fluids on the surface, a less controllable process that results in nonuniformly distributed SNs. Yang and Duisterwinkel presented a seminal work on the exploitation of SNs produced by SWE to remove NPs (i.e., polystyrene) from a surface.^8^ SWE was tested for ethanol and isopropanol. The surface was visualized before SWE using scanning electron microscopy and AFM as shown in Figure 1b)i. Multiple applications of SWE resulted in an improved PRE up to 90% which was associated with the cleaning action of SNs (Figure 1b)iii).^8^ Recently, the impact of SWE on SNs size and density has been well characterized as a function of the SWE’exchange rate, paving the way for controllable and repeatable application of SNs for cleaning purposes.^43^

However, the cleaning efficiency depends also on NPs’s characteristics such as pH, surface charge, and their physical/chemical nature. The role of pH was recently investigated by depositing nonacidic and acidic polystyrene latex nanoparticles on silicon wafers.^44^ NPs with acidic pH could not be removed after SWE. In contrast, the PRE for nonacidic nanoparticles was in the range of 80 to 90%.^44^

To verify if SNs generated by SWE could remove NPs of a different type, we employed silicon nitride NPs (Si_3_N_4_–NPs)with nominal size lower than 50 nm (Sigma- Aldrich, Merck). Before deposition, the NPs are dispersed in an NaOH solution (1:5 proportion, alkaline pH). Then, the solution is centrifuged for 30 min at 5k rpm to select NPs of smaller sizes. Finally, the supernatant is spin coated onto the silicon wafer (spinning velocity ω = 5 × 10^3^ rpm). For the SWE, we used ethanol (99% purity) and Milli-Q water. We monitored the cleaning steps using an AFM in tapping mode(Cypher from Oxford Instruments). We employed HQ:NSC18/Al BS from MikroMash with a nominal tip radius smaller than 8 nm.

**Figure 2:**
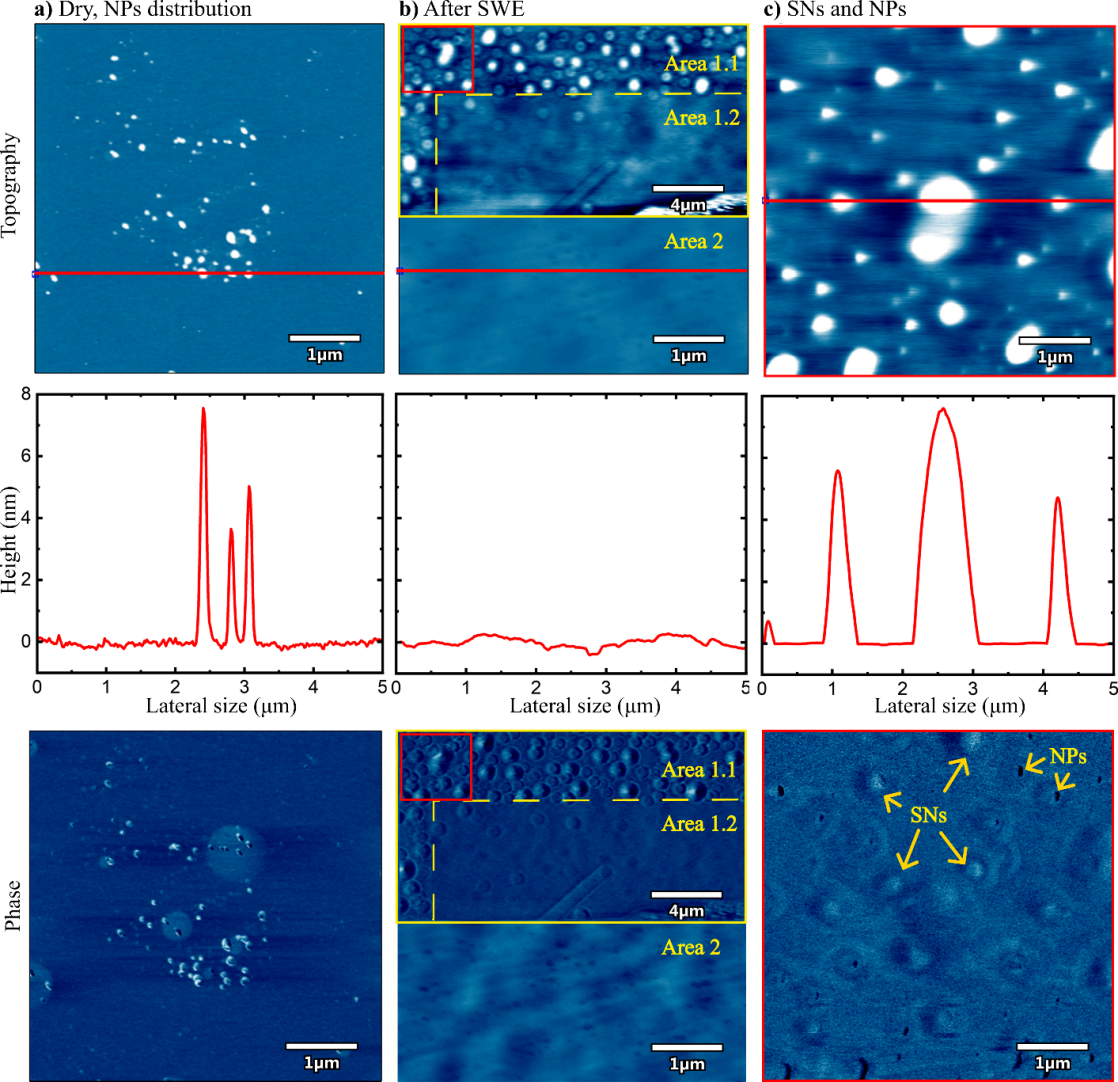
Si_3_N_4_–NPs removed from a silicon wafer after SWE. The first row shows the AFM topography; the second row presents section examples revealing topographical details (red line), and the third row shows the phase images. a) Si_3_N_4_–NPs imaged in air. b) Example of two surfaces after SWE. Area 1 (yellow box) is split in Area 1.1 highly populated by SNs and Area 1.2 free of nano objects, that is high PRE. Similarly, Area 2 does not present nano objects. c) Zoom in the details of the red box in b). SNs and NPs are distinguished by comparing the topography and phase signal.

Figure 2 illustrates the cleaning process composed of an image of NPs in dry conditions (a), the surface after SWE (b), and a detailed visualization on nano-objects (c). In Figure 2a, the topography shows a nonuniform spatial distribution of the distributed Si_3_N_4_–NPs with no regular shape. Phase imaging is an ideal method to support topographical images and distinguish Si_3_N_4_–NPs from SNs. The phase image in Figure 2a presents a low phase shift that complements the topographical information, suggesting that the objects interacting with the tip, i.e., Si_3_N_4_–NPs, possess similar mechanical properties.

Figure 2b shows the surface after SWE in two different Areas. Area 1 (scanning details: scan rate 3.47 Hz, set point 65.9 mV, integral gain 70.84) is divided into Area 1.1 highly populated with nano-objects and Area 1.2 where no nano-objects are observed. Similarly, Area 2 (scanning details: scan rate 1.17 Hz, set point 105.5 mV, integral gain 157.65) shows no nano features. To better understand the properties of the surface, in Figure 2c we show a zoom in Area 1.1 from Figure 2b (red box inset). After SWE, the topography reveals well-defined round cap shaped objects as expected from literature.^20,27,28^ The objects have heights between 2 and 10 nm and widths in the range from 500 to 1000 nm. The large phase shifts found after SWE indicate the tip interacting with softer objects, which we finally address as SNs (indicated by yellow arrows in Figure 2c).^20^ The presence of SNs depletes the interaction between the AFM tip and the surface as discussed elsewhere.^31,45,46^ Thus, Area 1.2 and Area 2 from Figure 2b are zones of high interaction (high adhesion) between the AFM tip and the surface, namely, of a high PRE.

## Discussion and Perspective

In order to critically discuss the cleaning ability of SNs and provide a perspective on the mechanisms of particle detachment, we discuss what is the interface of an isolated nanobubble and then how the nanobubble-nanobubble interaction can be described.

### What Is the Interface of SNs?

SNs possess a permeable interface which allows gas species and alcohol to exchange and interact with the environment, that is, the surface, the bulk, or the NPs. A strong experimental evidence comes from AFM and attenuated total reflection Fourier transform infrared, which allow to depict the mobility of gases across the SN’s interface, confirming its permeability.^47^ This result sustains the TAO model, where a influx/outflux regulated by hydrophobic forces is driving the long-term stability of SNs in saturated and undersaturated conditions.^32,33^

The gas–water interface, characterized for microbubbles, possesses a negative zeta potential (ζ-potential) stable over a wide pH range.^48^ Moreover, for microbubbles, it is demonstrated that alcohol molecules, coming as a result of the SWE, locally change the structure of the interface, affecting the ζ-potential. At the nanoscale, changes in the ζ-potential can be accessed by investigating isolated nanobubbles dispersed in a solvent.

### Nanobubble–Nanobubble Interaction

**Figure 3:**
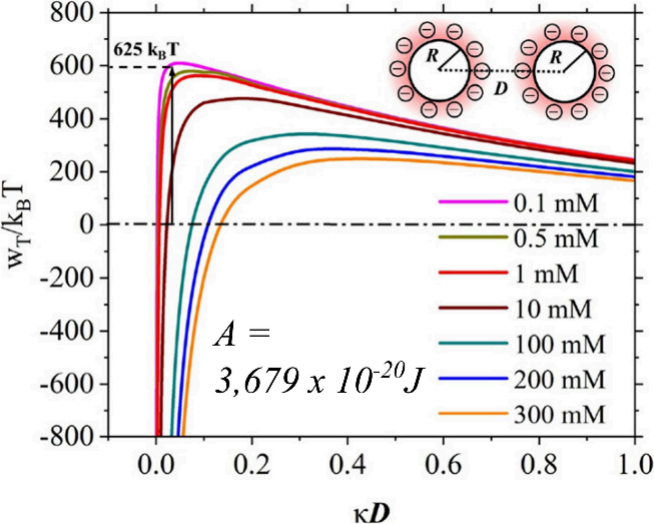
Total interaction potential between two nanobubbles in different electrolyte concentrations (NaCl), with Hamacker constant indicated (A). Adapted with permission from ref (46). Copyright 2023 American Chemical Society.

The stability and dynamics of a nanobubble dispersion can be investigated by imposing oscillatory pressure fluctuations followed by salting-out effects in water or electrolytes. Dynamic light scattering confirms the negative sign in ζ-potential of nanobubbles. Moreover, it shows that ζ-potential depends on the electrolyte valency and concentration (e.g., ζ-potential is −24 mV at 5 mM NaCl).^46^ By considering a Poisson distribution of ions around the single nanobubble (with nanobubbles described in the TAO model), the contribution of ζ-potential and surface tension to the nanobubble stability can be described. Specifically, the interplay of ions across the nanobubbles interface generates an EDL acting in synergy with the interfacial structural deformation provided by the alcohol molecules. If non-DLVO forces are excluded, Figure 3 depicts the total interaction potential *w*_T_ normalized by *k*_B_T at different electrolyte concentrations (NaCl), and plotted against the interspacing distance *κD*. If the distance *D* between nanobubbles, normalized by the Debye length κ^–1^, becomes small (*κD* < 0.5), VdW attractive forces will start contributing to the overall interaction, resulting in an Hamacker constant *A* = 3.679 ^–20^ J.^46^

Charging nanobubbles via ultrasound demonstrated a variation in electrokinetic surface charge with electrolyte concentration, which implies that ions are highly active at the nanobubble’s interface. The latest finding on the role of alcohol molecules and ion clouds shed light on the nanobubble’s interface. However, the theoretical description of the ion cloud activity presents geometrical limitations (Poisson axial approximation for the electrostatic potential) that cannot be directly transposed to SNs studies. A future challenge in the field is elaborating a novel description of the electrostatic potential adequate for SNs’ geometry.

### Are Surface Nanobubbles Responsible for Surface Cleaning?

In 2008 bulk nanobubbles were found to contribute to particle-surface adhesion rather than detachment in aqueous solution due to weak depletion forces.^45^ Nevertheless, in the past decade, nanobubbles (both from bulk or surface) have been associated with cleaning properties. Thus, the short answer to the section title is we still do not know. The experimental and theoretical studies described in the first section and the current understanding on nanobubble-nanobubble interaction do not clarify the question.

The way how the cleaning effects of SNs has been described in AFM experiments so far is basically a form of syllogism. (Note: Example of syllogism: it is true that all dogs are animals and it is true that all dogs have four legs. Therefore, all of the animals have four legs.) Let us consider NPs distributed on a silicon wafer (S) in air. After the application of SWE, none or only a few NPs remain on S, but SNs appear. Then, given that SNs are visualized after the cleaning step, the claimed logical conclusion is that SNs are responsible of cleaning. However, what the scientific evidence allow us to assert is only that SWE generates SNs *and* cleans the surface. In contrast, there is no experimental proof or theoretical verification to support that SNs act against NPs’ adhesion, promoting their detachment. This is the major point we want to stress in this perspective and, in our opinion, the major future challenge in the field.

### Possible Cleaning Mechanisms

**Figure 4:**
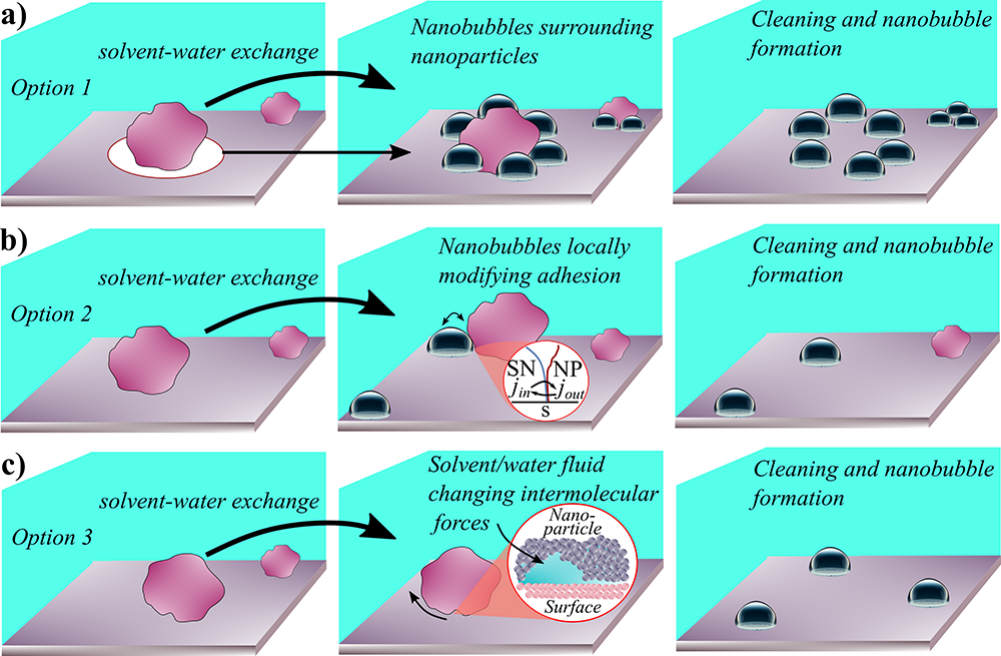
Proposed hypothesis of nanoparticle detachment upon application of SWE. a) Nanoparticles promote the formation of SNs around them. SNs change the pinning forces of the nanoparticles. After the cleaning step, SNs remain in the position of the nanoparticle’s footprint. b) SNs form randomly on a surface. SNs and nanoparticles in contact define a confined region where transport of molecules vary the nanoparticle’s adhesion. SNs’ role is to provide molecules to change locally the adhesion. c) The nanoroughness of nanoparticles allows fluids from the SWE process to modify the adhesion force promoting detachment. SNs are generated as results of the SWE, but do not play a role in the cleaning process. In all three cases, the result is always the removal of nanoparticles and observation of SNs.

In Table 1, we detail the possible force contributions in a liquid environment that would enable detachment of NPs. In the last column, we list our perspective in terms of the role of SNs to each force. As an indication for future research pathways, we present in Figure 4 three hypothetical mechanisms that may take place at the SNs/NP/S interface.

**Table 1:** Forces Participating in NPs Detachment and the Role of SNs

**Type of force**	**Characteristics**	**Role of SNs**
**VdW repulsion**	Arise between dissimilar bodies interacting in a medium at short relative distance, when their electron clouds overlap	SNs and/or SWE may initiate VdW repulsion in confined scenarios (e.g., Figure 4c).
**Buoyancy force**	Arise when a net upward force is generated in a fluid system (e.g., Archimedes’ principle)	The gradient in pressure between the gas enriched system that are SNs and NPs may promote detachment mechanisms.
**Quantum mechanical effects** (e.g., covalent bonding, steric repulsion)	Electron repulsion (Pauli’s principle) defining orbital overlap and spatial arrangement at the molecular level	The nanoscale confinement defined in the SNs-NPs-S system (Figure 4 b and c) can promote quantum effects.
**Electrostatic forces** (Coulombic forces, charge-exchange interaction)	Arise depending on surface charge distribution	SNs have negative ζ-potential which dictates their electrostatic interaction during the detachment mechanism.
**Solvation forces** (e.g., oscillatory force, structural force, hydration force)	Monotonically repulsive forces arising in solvent–solute systems, and participating in the adhesion mechanisms of molecules onto a surface.	SWE initiate these forces that may result in SNs formation but also be part of detachment mechanisms (Figure 4c).
**Nonequilibrium forces** (e.g., hydrodynamic forces, viscous forces, friction forces)	Energy-dissipating forces occurring during relative motion of surfaces or bodies	The growth and evolution of SNs in the proximity of NPs (see Figure 4a,b) is a dynamics systems that exchange energy (e.g., nanofriction), which may affect the adhesion of NPs on S.
**Entropic forces** (e.g., osmotic repulsion, double-layer force, thermal fluctuation force, undulation force, interface protrusion force)	These forces are based on the tendency of molecules to maximize entropy.	Fluctuations due to thermal effects or interface protrusion forces are designing SNs’ interfaces, ultimately changing their surface energy and interaction in the SNs-NPs-S system.

The first hypothesis is that SNs are likely formed around the nanoparticles. This phenomenon has been already discussed for bulk nanobubbles and SNs in nanotranches (Figure 1c).^8,35,41^ By generalizing this concept, we can hypothesize that the presence of NPs may act as an accumulator of SNs. Then, SNs might change the adhesion by redefining locally NPs’ pinning forces and consequently induce a detachment. The drawback of this hypothesis is, given the proven stability of SNs, that one must find a distribution of SNs around the NPs’ footprint (Figure 4a, third panel). Such a scenario has never been observed experimentally or computationally so far.

The second hypothesis does not require SNs to be formed around NPs, but rather to nucleate randomly on the surface. Moreover, it requires one to approach the problem from an atomistic point of view to verify the diffusion dynamics of gas and liquid molecules in the confined space defined by SNs and the NPs’ roughness. Some of the forces listed in Table 1 (e.g., VdW repulsion or quantum mechanical effects) are included since we suggest to look at the SNs-NP-S scenario as a confinement problem. The short-range interaction at the SNs/NP/S interface is driven by ion and molecules exchange. As illustrated in Figure 4b, the transport of alcohol molecules in the confined region, affects the DLVO interaction, possibly reducing the adhesion force and promoting detachment. In such a configuration, the role of SNs is still key, functioning as a local provider of molecules stored within its interface. Such a scenario will need to be approached with experiments and simulations to adequately describe the complexity in confinement. SFA is a technique that can provide insights about how molecules interact in confinement, such as EDL overlay.^29,49,50^

The third hypothesis presents SNs not as the catalyst of the cleaning process but rather as a byproduct. This possibility should be considered until proven differently. Intermolecular forces between a nanoparticle and a surface change during SWE regardless of the participation of SNs in the process.^7^ Indeed, when SWE takes place, fluids can travel underneath NPs (we call it at this point nanochannels) due to the nanoroughness. This diffusion of solvent (e.g., alcohol, water, ...) may strongly reduce molecular attraction forces such as VdW or electrostatic forces between NPs and S. This scenario leads to reduction of active adhesion forces, resulting in NPs’ detachment (see Table 1). Still, SNs will be formed because of SWE, in line with the literature (see Figure 1), but they would be simply a byproduct of the entire process, not key to the cleaning step. Verifying this hypothesis is extremely complex as requires visualizing the instantaneous SNs-NPs interaction. We believe this is a relevant experimental challenge.

## Conclusion

In this Perspective, we provide a discussion on the fundamental question if surface nanobubbles (SNs) promote detachment of nanoparticles (NPs) from a surface (S). After a brief review on what is known in literature (experimental, theoretical, atomistic investigation) in terms of SNs formation and growth (Section 1), we present the current understanding of cleaning efficiency of silicon wafer based on SWE and SNs, providing further information about removal of silicon nitride NPs via SWE (Section 2). Then, we propose a critical discussion and three possible hypotheses to describe the SNs-NPs-S scenario (Figure 4).

Specifically, in the third hypothesis, we propose that SNs might not be key to promote NPs cleaning, but rather a byproduct formed after SWE. We believe that verifying this hypothesis is relevant in the field and could help shedding light on confined phenomena with a large scope in cleaning technologies.

Finally, we list in Table 1 the forces that SNs may promote during SWE and propose that NPs removal is approached as a confinement question, where the DLVO interaction driven by the EDL overlay can help in expanding the theory. AFM and SFA appear to be ideal experimental approaches to verify the confinement hypothesis. MD simulations are further needed to understand how ions at the nanobubble interface behave across an electrolyte in a volume defined by the nanoparticle’s roughness (confined region).

To conclude, clarifying the nanoparticle detachment mechanism during SWE can pave the way toward effective cleaning at the nanoscale, with large impact in relevant industrial sectors such as semiconductor industry.
